# Distribution of hepatitis c virus (hcv) genotypes in patients with chronic infection from Rondônia, Brazil

**DOI:** 10.1186/1743-422X-8-165

**Published:** 2011-04-12

**Authors:** Deusilene S Vieira, Mónica V Alvarado-Mora, Lívia Botelho, Flair J Carrilho, João RR Pinho, Juan M Salcedo

**Affiliations:** 1 Laboratory of Tropical Gastroenterology and Hepatology, São Paulo Institute of Tropical Medicine and Department of Gastroenterology, School of Medicine, University of São Paulo, São Paulo SP, Brazil; 2 Oswaldo Cruz Foundation, Fiocruz Noroeste. Porto Velho, RO, Brazil

## Abstract

**Background:**

Hepatitis C virus (HCV) is an important human pathogen affecting around 3% of the human population. In Brazil, it is estimated that there are approximately 2 to 3 million HCV chronic carriers. There are few reports of HCV prevalence in Rondônia State (RO), but it was estimated in 9.7% from 1999 to 2005. The aim of this study was to characterize HCV genotypes in 58 chronic HCV infected patients from Porto Velho, Rondônia (RO), Brazil.

**Methods:**

A fragment of 380 bp of NS5B region was amplified by nested PCR for genotyping analysis. Viral sequences were characterized by phylogenetic analysis using reference sequences obtained from the GenBank (n = 173). Sequences were aligned using Muscle software and edited in the SE-AL software. Phylogenetic analyses were conducted using Bayesian Markov chain Monte Carlo simulation (MCMC) to obtain the MCC tree using BEAST v.1.5.3.

**Results:**

From 58 anti-HCV positive samples, 22 were positive to the NS5B fragment and successfully sequenced. Genotype 1b was the most prevalent in this population (50%), followed by 1a (27.2%), 2b (13.6%) and 3a (9.0%).

**Conclusions:**

This study is the first report of HCV genotypes from Rondônia State and subtype 1b was found to be the most prevalent. This subtype is mostly found among people who have a previous history of blood transfusion but more detailed studies with a larger number of patients are necessary to understand the HCV dynamics in the population of Rondônia State, Brazil.

## Background

Hepatitis C virus (HCV) infects around 170 million people, 3% of the world population, and is considered a worldwide public health problem [1,2]. The HCV genome is a 9.4 Kb single stranded RNA sequence with two untranslated regions at both ends (5 'UTR and 3' UTR) [3]. This coding region contains a single open reading frame encoding a polyprotein of approximately 3,000 amino acids that originate at least 10 viral gene products (C, E1, E2, p7, NS2, NS3, NS4A, NS4B, NS5A, NS5B) [4,5].

The virus represents the genus *Hepacivirus *of the family *Flaviviridae *[6] and it is classified into six major genotypes (1 to 6) and more than 80 subtypes. HCV genotypes may show extensive subtype diversity in some regions of the world, representing the spreading of this epidemic [7,8]. These genotypes differ by 31 to 34% in their nucleotide sequence and by around 30% in their amino acid sequence. Accurate HCV genotyping can be used for predicting response to anti-viral therapy, as genotypes 1 and 4 are less likely than genotypes 2 and 3 to respond to interferon [9].

Molecular tests are essential for confirmation of persistent HCV infection and to monitor and verify the success or failure of therapy. The advantages of these tests include the possibility of early diagnosis in acute viral infection, diagnosis of infection in patients unable to mount antibody response, and confirmation of active infection [10]. Besides its use for determining the type or duration of HCV, HCV genotyping is useful epidemiological studies, as genotypes vary among geographical regions and among different risk groups [11,12]. Sequencing of the NS5B has been standardized and used for identification of HCV subtypes, as the region contains subtype-specific motifs, and it is also appropriate for epidemiological applications [13].

In Brazil, it has been estimated that around 1.5% of the Brazilian population (> 2.5 million people) is anti-HCV positive [14,15]. Distribution of cases of chronic hepatitis C by transmission routes from 1998 to 2006 showed 21% of cases associated with intravenous drug use and 16% with blood transfusion, but in 40% of cases there was not any known risk factor [16]. An extensive review on HCV infection data in Brazil showed the following prevalence in healthy adults and/or blood donors in the different Brazilian regions: 0.9 to 2.4% (North), 1.7 to 3.4% (Northeast), 1.0 to 1.4% (Middle West), 0.8 to 2.8% (Southeast) and 1.1 to 2.1% (South) [17].

Rondônia (RO) State, in North-Western Brazil, has characteristics of a highly endemic region for viral hepatitis. A study carried out with the local population in the Madeira River at Porto Velho-RO showed the prevalence of hepatitis C reached 7.4% [18]. Ferrari et al. [19] investigated HCV seroprevalence among Karitiana Indians living in Rondônia and found that the HCV prevalence was 1.7%. According to the Brazilian Ministry of Health, during the period from 1999 to 2005, 1831 cases of hepatitis C were confirmed in the North Region and 400 (21.8%) of these cases were from Rondônia [18].

Recently, we reported the genotype distribution of HBV in Rondônia [20] and the present study is the first report on HCV distribution in this state. The aim of this study was to characterize the HCV genotypes circulating in Rondônia State (RO), Brazil.

## Methods

### Study Population

**Figure 1:**
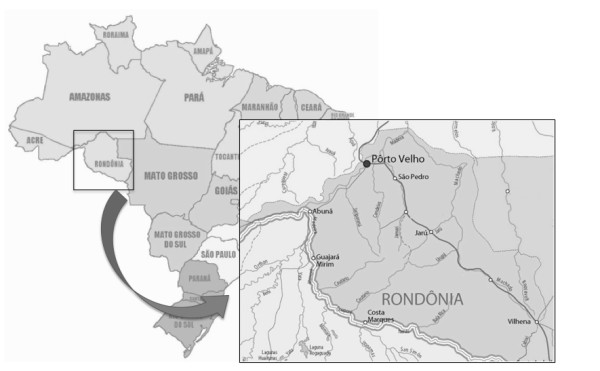
**Geographical localization of Rondônia state, Brazil**.

This study was carried out in the state of Rondônia, Brazil (Figure 1) and included 58 anti-HCV positive patients (29 males, 29 females, age ranging from 27 to 85 years old). All patients signed a consent form for this study. The samples were collected between January 2007 and October 2009 in the Oswaldo Cruz Foundation, Fiocruz Noroeste, Porto Velho-RO, Brazil. Data on previous blood transfusion and presence of liver cirrhosis or fibrosis were obtained from the review of each patient medical data.

### HCV RNA extraction

HCV-RNA extraction was carried out from 140 μL of serum using QIAamp^® ^Viral RNA Kit (QIAGEN, Valencia, CA, USA), following the manufacturer's instructions. The synthesis of the complementary DNA (cDNA) was made immediately after the RNA extraction. To avoid false-positive results, rigorous procedures proposed for nucleic acid amplification diagnostic techniques were followed [21].

### Synthesis of the complementary DNA (cDNA)

The reverse transcriptase reaction was performed using the enzyme Reverse Transcriptase of *Moloney Murine Leukemia Virus *and random primers. The final volume of the reaction was 60 μL with the following concentrations: 50 mM Tris-HCl (pH = 8.3), 75 mM KCl, 3 mM MgCl_2_, 10 mM DTT, 0.5 mM each dNTP (10 mM), 450 ng random primers, 30 units RNAse enzyme inhibitor (RNase OUT™) and 300 units M-MLV. Samples were submitted to the following temperature cycles: 70°C for 10 minutes, 25°C for 15 minutes, 37°C for 60 minutes and 95°C for 15 minutes in a thermocycler (Eppendorf Mastercycler ^®^, Eppendorf, Hamburg, Germany).

### Polymerase Chain Reaction (PCR)

Polymerase Chain Reactions (PCR) were carried out in two stages, first and second PCR, aiming to increase the reaction sensitivity. The NS5B (380 bp) region was amplified for genotyping analysis. Amplification primers and cycling conditions are previously described [22,23]. Reactions were carried out in a final volume of 50 μL. The cDNA (5 μl) was added to 20 mM Tris-HCl (pH = 8.3), 50 mM KCl, 1.5 mM MgCl_2_, 0.2 mM each dNTP (10 mM), 0.4 pmol/μL of each primer, and 2.5 units *Platinum *Taq DNA polymerase. All the reagents used were from Invitrogen ™ Life Technologies, Carlsbad, CA, USA.

### HCV sequencing

Amplified cDNA was purified using ChargeSwitch^® ^PCR Clean-Up Kit. Sequencing was performed in an ABI Prism^® ^377 Automatic Sequencer (Applied Biosystems, Foster City, CA, USA) using dideoxy nucleoside triphosphates (ddNTPs) containing fluorescent markers (*Big Dye^® ^Terminator v3.1 Cycle Sequencing Ready Reaction kit *- Applied Biosystems, Foster City, CA, USA). The quality of each electropherogram was evaluated using the Phred-Phrap software [24,25] and consensus sequences were obtained by alignment of both sequenced strands (sense and antisense) using CAP3 software available at the web page *Eletropherogram quality analysis *http://asparagin.cenargen.embrapa.br/phph/.

### Phylogenetic Analysis

The HCV sequences were genotyped by phylogenetic reconstructions using reference NS5B sequences from each HCV subtype obtained from GenBank (n = 173). The sequences were aligned using Muscle software [26] and edited in the SE-AL program (available at http://tree.bio.ed.ac.uk/software/seal/). Phylogenetic analyses were conducted using the Markov Chain Monte Carlo (MCMC) simulation implemented in BEAST v.1.5.3 [27] by both relaxed uncorrelated log_normal _and relaxed uncorrelated exponential molecular clock using the best model of nucleotide substitution (GTR+G+I) obtained by MODELTEST [28]. Twenty million generations were run to obtain the convergence of parameters. The maximum clade credibility (MCC) tree was obtained from summarizing the substitution trees and then it was removed 10% of burn-in using Tree Annotator v.1.5.3 [27].

## Results and Discussion

Among the 58 positive anti-HCV samples, only 22 were positive by nested PCR for NS5B region.

**Figure 2:**
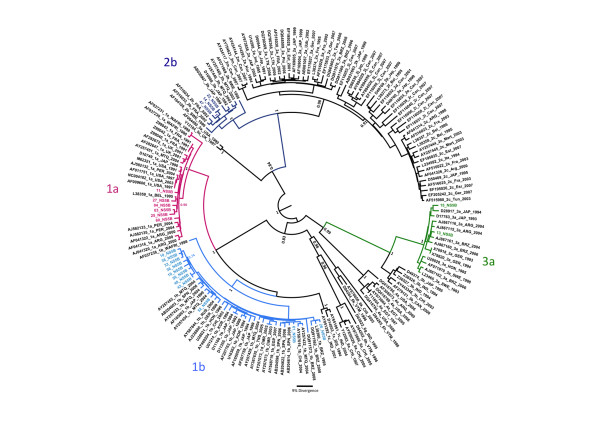
**The maximum clade credibility (MCC) tree was estimated by Bayesian analysis of 170 NS5B sequences with 313 bp of Hepatitis C virus strains**. The posterior probabilities of the key nodes are depicted above the respective nodes. The twenty-two sequences from Rondônia were highlighted: Six sequences subtype 1a (red), eleven sequences subtype 1b (blue), three sequenced subtype 2b (dark blue) and two sequences subtype 3a (green).

After sequencing, the phylogenetic analyses in this study showed that subtype 1b was the most prevalent in this population (50%). Also, the subtypes 1a (27.3%), 2b (13.6%) and 3a (9,0%) were detected in Rondônia State (Figure 2). Sequences were deposed in GenBank at accession numbers: HQ630386 - HQ630407.

Several studies were carried out to investigate the HCV distribution in the different states and different risk groups in Brazil [29-32]. Campiotto et al., [33] reported that genotype 1 was the most frequently genotype found in all regions of Brazil. Specifically, in the North region, genotype 1 was the most prevalent in Amazonas and Acre states (78.0 and 64.3%, respectively), followed by genotype 3. Genotype 2 was only observed in Amazonas state (7.1%). Also, genotype 1 predominates in the Southeastern Region of Brazil where the prevalence is around 70% in the states of Rio de Janeiro and São Paulo [34-36], and 84% in Minas Gerais [37]. In Salvador, Bahia state, genotype 3 was the most common genotype (53.3%), followed by genotypes 1 (40%) and 2 (6.7%) but, as the number of positive cases was small, other studies are necessary to confirm the high frequency of genotype 3 in Salvador [15]. Another study in Piauí state, Brazil, found 49.0% of subtype 3a, 26.0% of subtype 1a, 24.0% of subtype 1b and 1.0% of subtype 2b [38]. Furthermore, subtypes 1a (34.3%), 1b (30.0%) and 3a (25.7%) were frequently found in Brazilian blood donors, while 2b was rare, and 2a was not detected [39].

HCV subtypes found in Brazil represent several independent lineages probably originated from multiple introductions of each subtype into Brazil; analysis of Brazilian-specific clades provides a more accurate representation of the population dynamics of HCV in the country [40]. Furthermore, as Brazil is a large country with many different population backgrounds, a large variation in the frequencies of HCV genotypes is expected throughout its territory. In this study, as shown in Figure 2, phylogenetic analyses showed that subtypes 1a and 2b sequences from Rondônia clustered together but subtypes 1b and 3a have a more heterogeneous distribution along the tree. Since there are only 22 sequences reported in this study we cannot make any further inferences about HCV genetic variability.

Subtype 1b is mostly found among older members of the population who have a previous history of blood transfusion [41-43] but in this study the patients did not report previous blood transfusion. Also, subtype 1b is associated with a higher rate of chronic active hepatitis or cirrhosis, and with a poorer response to treatment with α-interferon than genotypes 2 or 3 [16]. In this study, all patients were naïve and two subtype 1a patients, as well as one subtype 1b patient had cirrhosis.

Genotype 3 has been associated with transmission through intravenous drug users what could have contributed to a large dissemination of this genotype [44]. The two subtype 3a female patients did not report previous drug injection and it is possible that this association is not valid in this region. However, the present study was not intended to analyze the association between HCV subtypes and routes of transmission.

In conclusion, this study is the first report on HCV genotypes from Rondônia state where subtype 1b was found to be the most prevalent. There was not any data from this region previously published. Rondônia state is one region where the population is growing faster in Brazil and it is relevant to analyze its health issues to allow the early implementation of health policies.

## Competing interests

The authors declare that they have no competing interests.

## Authors' contributions

DVS participated in the design of the study. MVAM conducted the phylogenetic and evolutionary analysis, drafted the manuscript and participated in its design and coordination. LB participated in the PCR amplification and sequencing process. FJC and JMS participated in the design of the study. JRRP participated in the elaboration of the manuscript. All authors read and approved the final manuscript.
